# Synthetic libraries of shark vNAR domains with different cysteine numbers within the CDR3

**DOI:** 10.1371/journal.pone.0213394

**Published:** 2019-06-17

**Authors:** Olivia Cabanillas-Bernal, Salvador Dueñas, Marta Ayala-Avila, Alexandra Rucavado, Teresa Escalante, Alexei F. Licea-Navarro

**Affiliations:** 1 Departamento de Innovación Biomédica, Centro de Investigación Científica y de Educación Superior de Ensenada (CICESE), Ensenada, Baja California, México; 2 Departamento de Farmacéuticos, Centro de Ingeniería Genética y Biotecnología (CIGB), La Habana, Cuba; 3 Instituto Clodomiro Picado, Facultad de Microbiología, Universidad de Costa Rica, San José, Costa Rica; Instituto Butantan, BRAZIL

## Abstract

The variable domain of New Antigen Receptors (vNAR) from sharks, present special characteristics in comparison to the conventional antibody molecules such as: small size (12–15 kDa), thermal and chemical stability and great tissue penetration, that makes them a good alternative source as therapeutic or diagnostic agents. Therefore, it is essential to improve techniques used for the development and selection of vNAR antibodies that recognize distinct antigens. The development of synthetic antibody libraries offers a fast option for the generation of antibodies with the desired characteristics. In this work three synthetic antibody libraries were constructed; without cysteines (Cys), with one Cys and with two Cys residues within its CDR3, with the objective of determining whether the presence or absence of Cys in the CDR3 favors the isolation of vNAR clones from a synthetic library. The libraries were validated selecting against six mammalian proteins. At least one vNAR was found for each of the antigens, and a clone coming from the library without Cys in the CDR3 was selected with all the antigens. *In vitro* angiogenesis assay with the isolated anti-VEGF antibodies, suggest that these vNARs are capable of inhibiting *in vitro* angiogenesis. *In silico* analysis of anti-VEGF antibodies showed that vNARs from synthetic libraries could rival antibodies with affinity maturation by *in silico* modeling.

## Introduction

Due to the important role of antibodies in the survival, neutralization and elimination of antigens, intensive efforts have been focused on understanding and exploiting their molecular specificity. With the objective of enhancing the properties of these molecules, different formats of antibodies have been developed, including Fab-fragments and single-chain Fv antibody fragments. These molecules are heterodimers with variable heavy-chain (VH) and variable light-chain (VL) domains. Advances in antibody development have resulted in the creation of single domain antibodies (sdAB) with only a VH or a VL domain, that maintain the original affinity for the antigen recognition [1–3].

In response to the tendency of reducing antibody formats to minimal fragments that retain their capacity for antigen union, two unique antibody isotypes have been found naturally containing a single domain for antigen binding. One of these antibody isoforms is found in the blood serum of Camelidae, these have been named heavy chain antibodies (HCAbs). These antibodies lack light chains and the constant domain 1 (CH1) [4, 5]. Another alternative format has been reported in cartilaginous fish such as sharks. This immunoglobulin called New Antigen Receptor (IgNAR) is a homodimer of two heavy chains joined by disulfide bonds, that lacks light chains. Each heavy chain contains five constant domains and a variable domain designated as vNAR, which is responsible for antigen recognition [6].

The vNAR domains possess special characteristics such as small size (12–15 kDa) and long and extended CDR3 (10–35 aa) [7–9]. These molecules have shown advantages over conventional antibody molecules like a higher thermal and chemical stability, better tissue penetration, resistance to a gastric pH, among others [10–15]. These unique characteristics make these antibodies ideal candidates for biotechnological and therapeutic uses.

**Fig 1:**
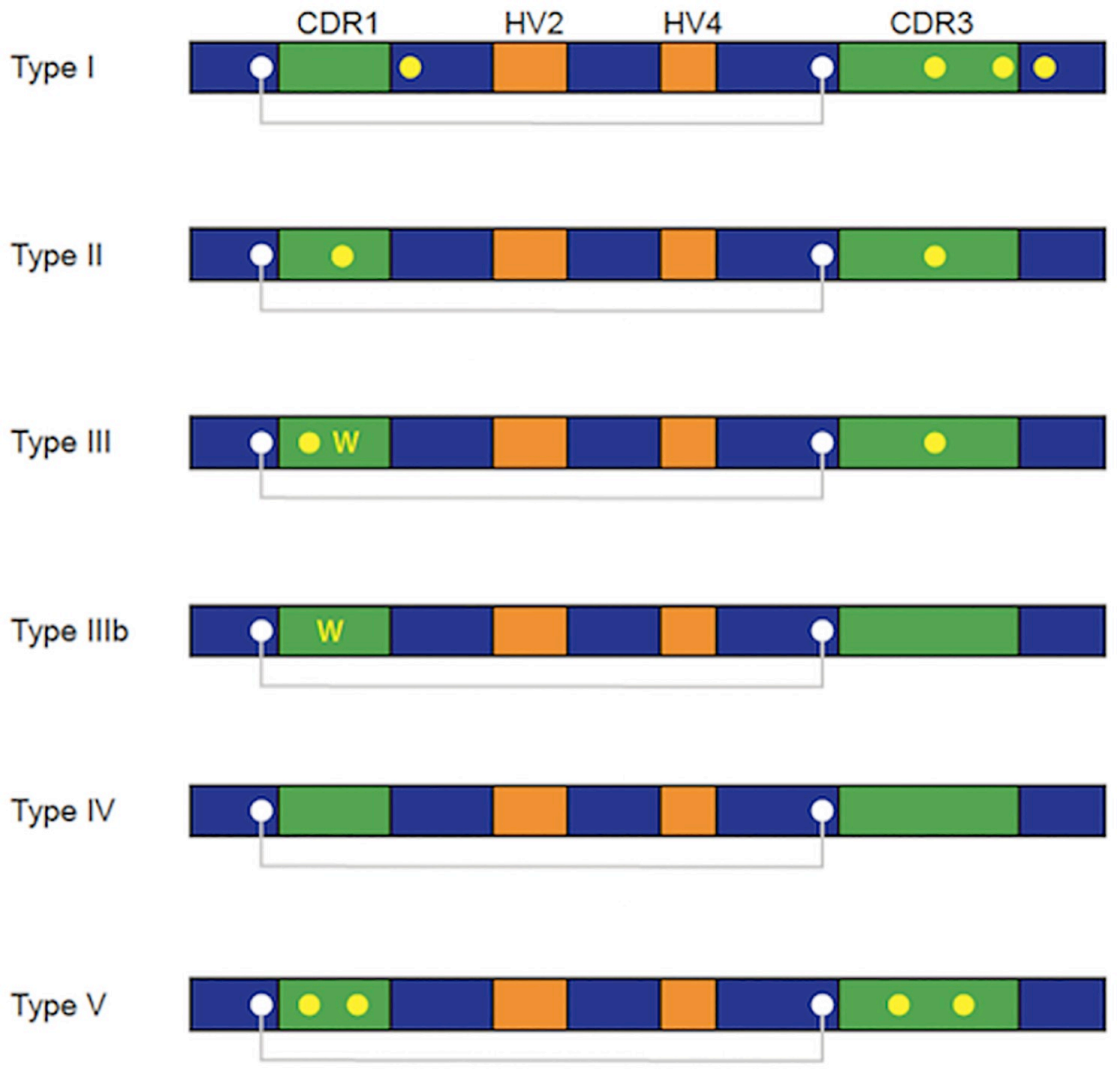
Distribution of the Cys pattern in the different vNAR types. According to the position and number of non-canonical Cys (yellow dots) in the variable domain, the IgNAR can be classified into different types. The canonical cysteine residues (white dots) and the conserved disulfide bond (Grey line) in all Variable (V) domains of the immunoglobulin family are labeled. Complementarity Determining Regions (CDR); Hypervariable loop (HV).

In accordance to the number and position of non- canonical cysteines (Cys) within the vNAR domain, their disulfide bond pattern and the time of appearance during the development of the shark, the IgNAR antibodies have been grouped in four principal types (Fig 1) [6, 8, 9, 16, 17]. Type I, with Cys residues in frameworks (FR) 2 and 4, and an even number of Cys in the CDR3. The Cys residues in FR 2 and 4 form an intraloop disulfide bond with the Cys pair in the CDR3. To date, this vNAR type has only been reported in the nurse shark (*Ginglymostoma cirratum*)[6, 9]. Type II vNAR present a simpler structure in comparison to type I, with Cys residues in the CDR1 and CDR3, which form an intra-molecular disulfide bond [6, 8, 9]. Type III vNAR is similar to type II, with non-canonical Cys in the CDR1 and CDR3 but it also possess a non-variable tryptophan (Trp) residue within the CDR1 which is not present in type I and II. Type III vNAR are found predominantly in newborn sharks (< 1 year), these have a constant size CDR3 and their diversity is limited, this type of vNAR probably serves as a first line of defense to pathogens before the maturation of the adult immune system [6, 9, 17]. The Type IV vNAR (also termed Type IIb) contain only two canonical Cys residues, and differs from the previous described vNAR types due to absence of non-canonic disulfide bonds [8,16,17]. Furthermore, another structurally different vNAR type named Type IIIb has been reported. It has a high similarity to type IV lacking the non-canonical Cys residues and the consequent non-canonical disulfide bonds, but as type III it has a conserved Trp residue at CDR1 [18, 19]. A different type of vNAR than those reported previously has been identified in the immune repertoire of the horn shark (*Heterodontus francisci*), and to our knowledge, in this work we present the first description of this vNAR antibody. We suggest this new type as type V, which has a pair of non-canonical Cys in the CDR3 and an additional pair of Cys in the CDR1 (unlike type I, that presents a pair of Cys in the FR2 and FR4).

Important advantages of synthetic libraries over the natural libraries are that they avoid the necessity of animal immunization, allow the relatively easy and rapid generation of recombinant antibodies with a specificity for practically any antigen [8,20,21] and also allow the incorporation of desired characteristics in the designs to allow relatively easy maturation of affinity [21, 22].

In this work, three synthetic antibody libraries were constructed from three different frameworks of vNAR antibodies; without cysteine, with one or two Cys residues within its CDR3, using the Kunkel mutagenesis technique [23], with the objective of determining if the presence or absence of Cys in the CDR3 favors the isolation of vNARs against a specific antigen. Phage display was carried out against six different mammalian proteins: Aquaporin 1 (AQP1), vascular endothelial growth factor (VEGF), basic fibroblast growth factor (FGF-2), leukemia inhibitory factor (LIF), carcinoembryonic antigen (CEA) and glycophorin A (GYPA). At least one vNAR was found for each of the antigens, and a clone isolated from the library without Cys at CDR3 was selected for all the antigens. To evaluate if the isolated antibodies from the synthetic libraries were functional, anti-VEGF (VS1-20 and VS0-4) antibodies were employed. The *in vitro* anti-angiogenic effect of VS1-20 and VS0-4 was evaluated and results suggest that these antibodies are capable of inhibiting angiogenesis in a three-dimensional model based on endothelial cell spheroids. *In silico* analysis of anti-VEGF antibodies showed that vNARs from synthetic libraries could rival vNARs whose affinity had been improved through *in silico* modeling, with *in vitro* and *in vivo* anti-angiogenic effect previously reported [24]. This study also presents the first report of the construction of synthetic libraries of vNAR domains, using frameworks with three different number of Cys at their CDR3.

## Materials and methods

### Ethics statement

This study was conducted in strict accordance with the WHO international operational guidelines for the ethics committees that review biomedical research and with the recommendations of the "CICESE Bioethics Commission". Protocol approved on 09/01/2016.

### Phagemid and *Escherichia coli* strains

*Escherichia coli* (*E*. *coli*) strain CJ236 (New England BioLabs) was used to generate the uracil-containing single stranded DNA (ssDNA). A TG-1 strain of *E*. *coli* (Lucigen, Madison, WI) was used to select the mutant double-stranded DNA (dsDNA). BL21 (DE3) strain of *E*. *coli* (New England BioLabs) was used for protein expression. The bacteriophage M13K07 (Invitrogen) and pComb3X phagemid [25] was used.

### Selection of vNAR frameworks

**Fig 2:**
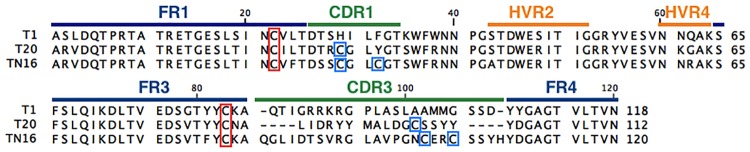
Sequences of vNAR domains used as frameworks for libraries construction. T1 without Cys, T20 with one Cys and TN16 with two Cys residues within its CDR3. The different regions of the vNAR are labeled. FR: Framework, CDR: complementarity-determining region, HVR: Hypervariable region. Canonical Cys are enclosed in red. Non canonical Cys are enclosed in blue.

Three vNAR protein sequences (previously isolated by our research group) were selected as a framework for the construction of the synthetic libraries (Fig 2). The selection was carried out considering the number of Cys within the CDR3. The selected frameworks were T1, T20 and TN16 with 0, 1 and 2 Cys in their CDR3, respectively. These frameworks come from two immune cDNA libraries of horn shark antibodies. T1 and T20 were isolated from an immune library against transforming growth factor beta (TGF-β), whereas TN16 was isolated from an immune library against tumoral necrosis factor alpha (TNF-α).

**Fig 3:**
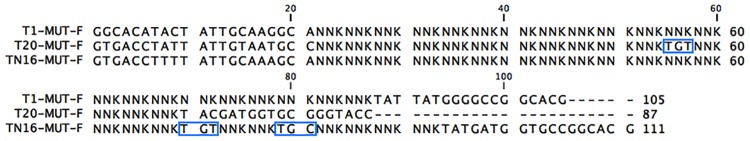
Mutagenic oligonucleotides used to diversify the CDR3. The oligonucleotides T1-MUT-F, T20-MUT-F and TN16-MUT-F were used with framework T1, T20 and TN16 respectively. With the exception of the cysteine-encoding codons (enclosed in blue), all of the other codons at CDR3 encode NNK. The length of the CDR3 of each framework was respected.

To improve the efficiency during the selection of libraries, the vNAR genes were designed to include three stop codons (not amber) after the canonical Cys of FR3 (just before the CDR3). The stop codons in the templates are intended to be eliminated with the mutagenic oligonucleotides and prevent the display of the original domains. The sequences of the three genes were optimized for codon usage in *E*. *coli* (Integrated DNA Technologies, IDT). One mutagenic oligonucleotide was designed for each framework (Fig 3), respecting the length and the number of Cys within the CDR3. With the exception of Cys encoding codons, the rest of the codons encode NNK (where N is an equimolar mixture of A, G, C, and T, and K is an equimolar mixture of G and T). Six to seven triplets were left before and after the CDR3 for overlap during the alignment reaction.

### Uracilated single stranded DNA production

Chemocompetent CJ236 cells were transformed with the pComb3X carrying the template sequences. The transformed cells were grown in a Petri dish containing 2xYT medium (2x Yeast extract and Tryptone: (1.6% (w/v) tryptone, 1.0% (w/v) yeast extract, 0.5% (w/v) NaCl and 1.5% (w/v) agar, supplemented with 100 μg/mL carbenicillin (carbenicillin was used in a constant concentration of 100 μg/mL) and 2% glucose and incubated overnight (ON) at 37 °C. Ten colonies were randomly selected and each colony was inoculated into 10 mL of 2xYT medium containing carbenicillin, 5 μg/mL chloramphenicol and 2% glucose. The cultures were incubated at 37 °C with constant agitation (250 rpm) until an optical density at 600 nm (OD_600_) of 0.4 to 0.6 was reached. The 10 mL of the cultures were infected with 20 particles of helper phage M13K07 per cell. The cultures were incubated at 37 °C for 30 min with no agitation. The cells were pelleted (centrifuged at 3300 rpm, for 40 min) and resuspended in 40 mL of 2xYT medium containing carbenicillin, 50 μg/mL kanamycin, 0.25 μg/mL uridine and 2% glucose. The cultures were incubated at 37 °C, with agitation for 20 h. The cultures were centrifuged at 3500 rpm, for 15 min, at 4 °C. The supernatant (SN) was recovered and 1/5 of the volume of 20% PEG 8000/2.5 M NaCl solution was added, gently mixed and incubated on ice for 1 h to precipitate the phages. Phages was centrifuged at 3500 rpm for 15 min, at 4 °C and the SN discarded. Each phage pellet was resuspended in 0.5 mL of PBS. To remove the remaining bacteria, the solution was centrifuged at 10,000 rpm for 15 min. The SN was recovered, pooled and filtered through a 0.2 μM filter. The extraction of ssDNA from the phage was performed with the M13 QIAprep Spin kit (QIAGEN). The purified DNA was quantified in a nanodrop and evaluated on 1% agarose gel.

### Construction of the libraries by Kunkel mutagenesis

For the construction of the libraries, the Kunkel mutagenesis protocol was used with some modifications [23]. The mutagenic oligonucleotides were phosphorylated using the following reaction: 2 μL of 10 X TM buffer (0.5 M Tris, 0.1 M MgCl_2_, pH 7.5), 1 μL of 100 mM DTT, 11 μL of H_2_O, 2 μL of T4 Polynucleotide Kinase Enzyme (T4 PNK: 10 U/μL, New England BioLabs) and 2 μL of 330 ng/L mutagenic oligonucleotide. The reaction was incubated 1 h at 37 °C. The alignment reaction was then prepared by adding 20 μg of the previously prepared uracilated ssDNA, 20 μL of the phosphorylated mutagenic oligonucleotide, 25 μL of 10 X TM buffer and H_2_O was added for a final volume of 250 μL. The alignment was carried out in thermocycler using the following program: 3 min at 90 °C, 3 min at 50 °C and 5 min at 20 °C. The reaction was then placed on ice. The following mutant strand filler reaction was prepared: 25 μL of 10 mM ATP, 37.5 μL of 100 mM DTT, 62.5 μL of 10 mM dNTPs, 15 μL of 400 U/μL T4 ligase (New England BioLabs) and 7.5 μL of 10 U/μL T7 DNA polymerase (New England BioLabs). 60 μL of this reaction were taken and added to the 250 μL of the previous alignment reaction and was incubated ON at 20 °C. The filler reaction was evaluated on 1% agarose gel. The mutagenic dsDNA was purified using the QIAquick Gel Extraction Kit (QIAGEN). 80 μg of dsDNA was mixed with 700 μL of electrocompetent TG1 cells, divided into 4 electroporation cuvettes (0.2 cm) and electroporated (2.5 kV). Cells were recovered by washing each cuvette with 2.5 mL of 2xYT medium containing 2% glucose. The cell suspension was incubated at 37 °C, with agitation, for 1 h. Serial dilutions of the culture were prepared to estimate the size of the library. The culture was pelleted and grown in four petri dishes (60 mm) in 2xYT agar medium containing carbenicillin and 2% glucose and incubated at 37 °C ON. The bacteria were recovered by washing the plates with 2xYT medium containing carbenicillin and 2% glucose. Cells were used for plasmid DNA extraction using the PureLink Quick Plasmid Miniprep Kit (Thermo Fisher Scientific) and for preparing the phage library. Ten random colonies from each library were selected from serial dilutions and evaluated by PCR using Ompseq (5'-AAG ACA GCT ATC GCG ATT GCAG-3') and gback (5'-GCC CCC TTA TTA GCG TTT GCC ATC-3') oligonucleotides, the plasmid DNA of positive clones was extracted and sequenced (Seqxcel, San Diego, CA, USA) to corroborate the variability in the library. The diversity of the libraries was confirmed by sequencing an additional 60 colonies (Department of Biomedical Innovation, CICESE). Sequences were analyzed using the CLC Main Workbench, Version 7.6.4 (QIAGEN, Aarhus A/S). Ninety two clones were randomly selected from the dilutions, the phage from the individual clones was produced and used in a ELISA to estimate the percent of the library that displayed a complete vNAR, using an anti-HA tag antibody as capture antigen and the anti-M13-HRP antibody for detection.

Phages particles of the synthetic libraries were amplified (as described in uracilated single stranded DNA production section) from the transformed bacteria suspension and resuspended in sterile 1X PBS containing 20% glycerol.

### Selection of libraries against different antigens

Six antigens were used for the validation of the libraries: Aquaporin 1 (AQP1), vascular endothelial growth factor (VEGF), basic fibroblast growth factor (FGF-2), leukemia inhibitory factor (LIF), carcinoembryonic antigen (CEA) and glycophorin A (GYPA). Phage display was conducted as described by Barbas *et al*., 2001 [25]. In brief, 250 ng/well of antigen of interest was immobilized. Antigen concentration was reduced by 50% from round 1 to round 2, then maintained at the same concentration for the following rounds. Four rounds of selection were carried out. The stringency of selection was raised with an increment in the number of washes during each round with 1X PBS-0.05% Tween (PBST) (5, 10, 15 and 15 washes for round 1–4, respectively). 20–40 clones were selected randomly from rounds 3 and 4 and were evaluated by PCR using Ompseq and gback oligonucleotides or by phage ELISA. The plasmid of 10–20 positive clones from each library per antigen was isolated and sequenced.

### Expression of recombinant vNAR antibodies

Positive plasmids were transformed into electrocompetent *E*. *coli* BL21 (DE3) cells. A single colony of interest was placed in 5 mL of SB medium containing carbenicillin and was incubated ON at 37 °C with agitation. The ON culture was used to inoculate 500 mL SB medium containing carbenicillin and incubated at 37 °C with agitation until culture reached an OD_600_ = 0.6–0.8. Induction was performed with 1 mM IPTG (Sigma Aldrich) and incubating and additional 6 h. The culture was centrifuged at 4500 rpm for 15 min. The cell pellet was used for the periplasmic extraction of the protein according to QIAexpressionist manual (QIAGEN). For the protein purification the His Tag Protein Purification: Ni-NTA Agarose (QIAGEN) was used. The sample was passed three times through the column, and two washes of 10 mL were performed increasing the concentration of imidazole (20 mM to 50 mM). The protein was eluted in five fraction of 0.5 mL of elution buffer (with 250 mM imidazole). Fractions containing the vNAR protein was dialyzed in 1X PBS and quantified using the Micro BCA protein assay kit (Thermo Scientific).

### Antigen recognition by ELISA

Fifty μL of the periplasmic extract was added per well in triplicate and incubated ON at 4 °C. Wells were blocked with 150 μL/well of blocking solution for 1 h at 37 °C. The solution was removed and 50 μL/well of anti HA-HRP antibody (1:1000) (Roche) in 1% BSA was added and incubated for 1 h at 37 °C. The SN was discarded and the wells were washed three times with PBST. Fifty μL/well of TMB solution was added and incubated for 15 min at 37 °C. Fifty μl of stop solution (1N HCl) was added per well and its absorbance was determined at 450 nm using a plates reader. 1% BSA was use as a negative control.

To confirm which clones had the ability to recognize the target antigen 250 ng/well of the antigen was immobilized in triplicate and incubated ON at 4 °C. The wells were blocked as described above. The solution was removed and 50 μL/well of the recombinant vNAR was added and incubated at 37 °C for 1 h. The SN was discarded and the wells washed three times with PBST. A volume of 50 μL per well of anti HA-HRP antibody was added. From here on the methodology was developed as stated in the previous section.

The binding ability of vNARs with positive recognition for their target antigen, was evaluated against the original antigen of the frameworks used for the libraries by sandwich ELISA (duplicate test). In triplicate, 250 ng of purified vNARs were used as capture antibodies. After blocking the wells, 125 ng of recombinant human TGFβ (PeproTech) was added to each vNAR. The commercial anti-TGFβ mouse mAb ID11.16.8 (1:3000) (Bioxcell) was used to detect the capture antigen. The sandwich was detected by a goat anti-mouse IgG antibody conjugated to HRP (1:10,000) (Santa Cruz Biotechnology, Inc.). Finally, the ELISA was reveled with TMB, and absorbance was read at 450 nm. The commercial mAb ID11.16.8 was used as positive control to compare binding efficiency, and was performed in an indirect ELISA format.

### Western blot analysis

Tricine-SDS-PAGE methodology was performed as describe by Schägger and von Jagow [26]. The gel proteins were transferred to nitrocellulose membrane (BIORAD, 162–0115) at 200 mA, 1 h on a semidry system. The membrane was incubated for 2 h at room temperature (RT) and moderate agitation in blocking solution (5% skim milk in PBST). The membrane was then washed with PBST and incubated for 2 h, at RT with moderate agitation in a 1:1000 dilution of anti HA-HRP antibody in 1% skim milk in PBST. The membrane was washed with PBST and finally proteins were detected by colorimetry using Pierce DAB Substrate Kit (Thermo Scientific) or by chemiluminescence using Pierce ECL Western Blotting Substrate (Thermo Scientific).

### Three-dimensional *in vitro* angiogenesis assay: Endothelial cell spheroids model

*In vitro* angiogenesis was measured using an endothelial cell (EC) spheroid-based three-dimensional assay [27]. Human umbilical vein endothelial cells (HUVEC) (Cell Applications, Inc) were cultured in EC growth medium (ECGM) at 37 °C on a humidified incubator under an atmosphere of 5% CO_2_ in air; only cells cultured from passages 3–5 were used. HUVEC monolayers with an 80% confluence were trypsinized and resuspended in ECGM medium with 20% metocel, in a 400 cells/100 μL dilution, to generate spheroids of approximately 400 cells. The cell suspension was distributed in a 96 well U bottom non-adherent plate and incubated at 37 °C with 5% CO_2_ for 24 h to allow spheroids formation. The spheroids were recovered from the plates, centrifuged at 300 g for 10 min and were carefully resuspended in a collagen solution (2 mg/mL) at 4°C, pH 7.4, previously mixed with 1:1 metocel with 20% fetal serum bovine (FSB). The solution with the spheroids was quickly distributed in a non-adherent 24 well flat bottom plate; 1 mL of solution containing approximately 40 spheroids was deposited in each well and the plate was incubated at 37 °C for 30 min to allow the gelification of the collagen. The different treatments (100 μl) were then added: 1) basal medium (MB, control group); 2) VEGF (50 ng) + MB; 3) VEGF (50 ng) + VS1-20 antibody (10μg); 4) VEGF (50 ng) + VS0-4 antibody (10μg). In treatments with anti-VEGF antibody, vNAR antibodies were incubated for 30 min with VEGF, prior to application on the spheroids. The plate was incubated in a humidified incubator for 4 h at 37°C with 5% CO_2_. Furthermore, an additional 10 μg of antibody anti-VEGF (in 50μl of PBS) was added to the corresponding treatments and the plate was incubated for a total of 24 h. The spheroids were fixed by adding 1 ml of 10% formalin; images of 20 spheroids from each treatment and experiment (duplicate test) were captured using an inverted microscope (Olympus, Germany) and a digital image software (Image Pro). The *in vitro* angiogenesis was quantified digitally by measuring the cumulative length of the capillary-like sprouts that grew on each spheroid.

### Statistical analysis

Differences between treatment groups were tested by unpaired Student’s t test. The P-values <0.05 were considered of statistical significant.

### Homology modeling

The three-dimensional structures of VS0-4 and VS1-20 were predicted by homology-based modeling using MODELLER v.9.16 [28], through a strategy known as “Advanced Modeling.” BLAST-P was used to identify the consensus template structures for modeling. vNARs were modeled based on three distinct protein scaffolds of the different IgNAR proteins with more than 50% identity. The template PDB files downloaded from the Protein Data Bank (PDB), with PDB ID 4HGK, 2I26, and 2I24.

### Molecular dynamics—Simulated annealing strategy

The three-dimensional structure of the vNARs were refined by simulated annealing (SA) calculations with software named Nanoscale Molecular Dynamics (NAMD) [29], followed by analysis and visualization of the results using the molecular graphics software Visual Molecular Dynamics (VMD) [30] and MacPyMOL: PyMOL v1.7.4.4 Edu Enhanced for Mac OS X. For quality control purposes, Ramachandran plots of the vNAR obtained with PROCHECK server tool [31]. The simulations were performed in a water box as the solvent with periodical boundary conditions, where an NPT ensemble was assumed, a constant number of particles (N), constant isobaric (P) and isothermal (T) conditions. The pressure was set to 1 atm, and the temperature to 300 K. These conditions were coupled to annealing (heating) and relaxation (cooling) steps iteratively. After annealing and cooling, each mutated scaffold was subject to a molecular dynamics analysis at 300 K and, 1 atm during 50 ns. Analysis of atom trajectory coordinates and energies were written to disk every ten ps. The most thermodynamically stable protein conformation with the longest existence time was selected.

### Docking, protein-protein affinity and interaction surface predictions

To predict the possible binding site of VS0-4 and VS1-20 vNARs to VEGF, applied a protein-protein docking protocol, using the ClusPro web tool [32]. The obtained complexes were filtered, selecting those with good electrostatics and desolvation free energies for further clustering. For visualization and select of the better results using MacPyMOL: PyMOL v1.7.4.4 Edu Enhanced for Mac OS X. A complex with better binding affinity and orientation was chosen as possible VEGF-VS0-4 and VEGF-VS1-20 complexes. To identify predicted protein-protein interaction regions, we used the tool Peptidederive in the ROSIE server, with default settings. For the docking calculation, Peptidederive gave a plot with the possible protein-protein interactions residues involved in the interaction, ranked according to the Rosetta scoring function energy–Rosetta Energy Units (REU) [33, 34].

For a better interpretation of our results we use a positive control (V13_P98Y/VEGF) [24] to have an idea of the REU values obtained for the interaction with an *in silico*, *in vitro* an *in vivo* developed VNAR against VEGF.

### Analysis of the blocking activity of VS0-4 and V13_P98Y on the VEGF/VS1-20 interaction by competitive ELISA

The *in silico* models of VS1-20, VS0-4 and V13_P98Y, predicted the possible binding site of these antibodies to VEGF. The *in silico* analysis were corroborated by a competitive ELISA (duplicate test), to evaluate whether these antibodies have an overlapping epitope and could block each other’s binding to VEGF. The plates were coated with 250 ng of recombinant human VEGF (PeproTech) and blocked. Triplicate samples of 250 ng of soluble, purified VS0-4, V13_P98Y, VS1-20 (as a positive control), vNAR CV0-43 (as a negative control) or PBS were added. Then VS1-20 phage was added in a 10^−3^ dilution. The binding was detected by an anti-M13-HRP antibody (1:5000) (Sigma Aldrich). Finally, the ELISA was reveled with TMB, and absorbance was read at 450 nm.

## Results

### Construction of the synthetic vNAR libraries

Three vNAR sequences, previously isolated in our laboratory from the immune repertoire of the horn shark (*H*. *francisci*) were chosen as frameworks, considering the number of Cys that occurred naturally within the CDR3 of the vNAR. In Fig 2, the sequences of the selected frameworks are shown. T1 a type IV vNAR, T20 a type II vNAR and TN16 a vNAR similar to type I vNAR, with two Cys within the CDR3, but unlike type I that contain an additional pair of Cys at the FR2 and FR4, TN16 present a non-canonical Cys pair in the CDR1. This particular vNAR type has been proposed as type V. The CDR3 loop of the selected frameworks presented lengths of 22, 16 and 24 amino acids, respectively.

The mutant dsDNA was obtained (S1 Fig) by Kunkel mutagenesis and it was used to transform TG1 cells. Serial dilutions (10^−1^–10^−12^) were prepared from transformed cell to estimate the libraries sizes. The initial sizes of the obtained libraries were: 1.8x10^9^, 6.7x10^9^ and 5.7x10^11^, for T1, T20 and TN16 respectively.

Sequence analysis of ten clones from each library showed that for T1, seven of the analyzed sequences were new and different, for T20 and TN16 six sequences were completely new and different and four corresponded to the original sequence. Since T1, T20 and TN16 correspond to the name of the genes used as frameworks, from this point on the libraries were renamed as VS0, VS1 and VS2, respectively, the number within the name of the library, denotes the number of cysteins engineered into CDR3. Taking into consideration the percentage of variability obtained for each library (70% 60% and 60% for VS0, VS1 and VS2, respectively) a readjustment of the size of the library was performed. The final library sizes were: 1.2 x10^9^, 4.0 x10^9^ and 3.4 x10^11^, respectively. To confirm the diversity of the libraries, 60 additional colonies were sequenced. The sequences showed approximately 65% diversity, in agreement with the results obtained in the initial sequencing. To estimate the percentage of phages in the libraries that displayed a complete vNAR, 92 clones were randomly selected and were evaluated by phage ELISA. The percentage of clones displaying a complete vNAR was approximately 86%.

### Validation of the synthetic libraries

The validation of the libraries was conducted by selecting against six different mammalian proteins by phage display. Positive clones were isolated against all antigens. Table 1 presents the sequences of the CDR3 of several clones isolated against different antigens, the characteristic Cys of the CDR3 of each library is marked in grey.

**Table 1:** Examples of the CDR3 sequence of several antibodies selected against the different antigens. The bold type and underlined Q represent an amber codon replaced by glutamine. The characteristic Cys of the CDR3 of each library is marked in gray.

Library	Antibody	CDR3 sequence	Antigen
VS0	AQ-10	LTFS**Q**FNPWCSVVRWGVAFLVG	AQP1
	VS0-42	QPQDKDDDSLVQCVADGVMGFL	VEGF
	FGV0-10	SVMPWGYADWVGGS**Q**KGTAMGK	FGF-2
	CV0-47	SVLRGKYGWSLLSGGLPRRLGT	CEA
	CV0-91	GYGRLSILGFLFGHYGPRLHGA	CEA
	GPV0-2	KAVSDITLRMVSCAWQGSCPSMRR	GYPA
VS1	AQ-1	**Q**TGVEVPKFR**Q**CLWPG	AQP1
	VS1-7	DF**Q**AVWFVCECCRIRS	VEGF
	FGV1-27	PWLCSVGATVRCTLVM	FGF-2
	LIV1-16	PAQLGSRWGRLCWSHL	LIF
	CV1-65	EAGWGKFWNGWCKPAT	CEA
	GPV1-3	PWYRD**Q**LVLWLCWWWA	GYPA
VS2	VS2-5	DNFCGGVCSSVMV**Q**GLCLSCGKYY	VEGF
	LIV2-7	LGPYTDI**Q**WTEKLQAFCTTCWLSS	LIF
	LIV2-42	**Q**APDCGRTVRSKCWSFCHLCGLEV	LIF

The results of the selection against the different antigens are summarized in Table 2. Some of the sequences obtained through mutagenesis incorporated an amber stop codon in the CDR3. As expected, all the sequence isolated from library VS1 and VS2 maintained the characteristic Cys within the CDR3 (position 97 for VS1; 102 and 105 for VS2).

**Table 2:** Summary of the total clones isolated during the selection with each library and antigen. Clones with or without amber stop codons are listed.

	VS0		VS1		VS2	
	No amber	amber	No amber	amber	No amber	amber
AQP1	2	1	-	2	-	2
VEGF	2	2	3	6	-	1
FGF-2	2	4	3	6	-	-
LIF	7	5	2	5	-	2
CEA	11	-	3	2	-	-
GYPA	2	1	-	4	-	-

### Expression of the antibodies and antigen recognition by ELISA

**Fig 4:**
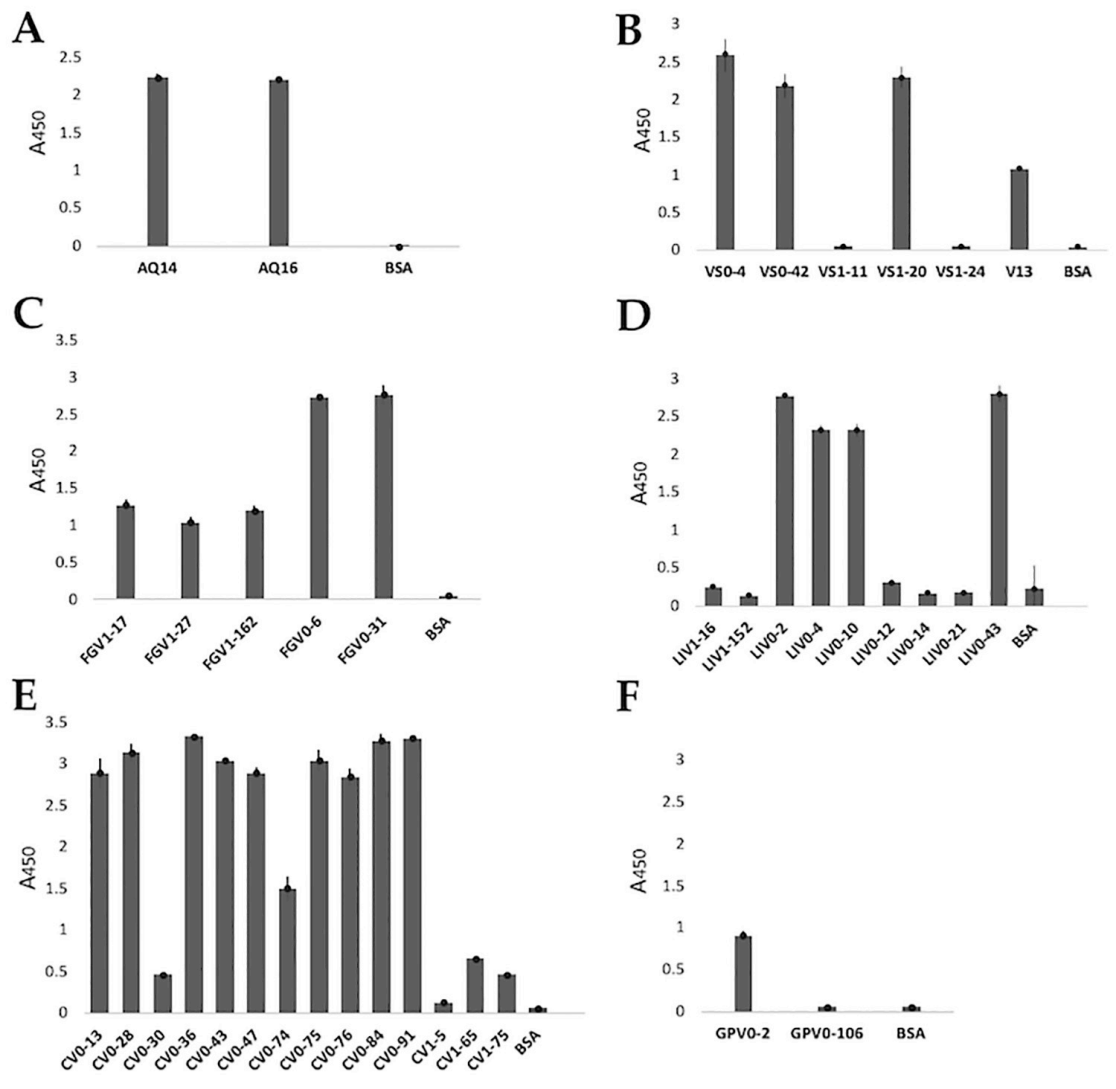
Evaluation of recombinant vNARs expression by ELISA. Protein expression was evaluated from the periplasmic extract. Antibodies with positive expression (absorbance at least twice that the negative control) were: (A) Anti-AQP1: AQ14 and AQ16; (B) Anti-VEGF: VS0-4, VS0-42 and VS1-20, V13 was used as positive control; (C) Anti- FGF-2: FGV1-17, FGV1-27, FGV1-162, FGV0-6 and FGV0-31; (D) Anti-LIF: LIV0-2, LIV0-4, LIV0-10 and LIV0-43; (E) Anti-CEA: CV0-13, CV0-28, CV0-36, CV0-43, CV0-47, CV0-74, CV0-75, CV0-76, CV0-84, CV0-91 and CV1-65; and (F) Anti-GYPA: GPV0-2. In all assays, BSA was used as negative expression control. A450: Absorbance at 450 nm.

For the expression analysis, the antibodies that did not present an amber stop codon in the CDR3 were evaluated, because these antibodies could be directly transformed in an expression strain such as *E*. *coli* BL21 (DE3). Antibodies that incorporated an amber codon in their CDR3 need an extra step for the substitution of this codon for a glutamine (Q). No expression was carried for the antibodies from the library VS2 because no new antibodies were found or the newly obtained sequences had an amber stop codon at CDR3. Recombinant protein expression was evaluated from the periplasmic extract, by ELISA (Fig 4). Positive expression levels were observed for 27 clones (up to 70% of the clones).

**Fig 5:**
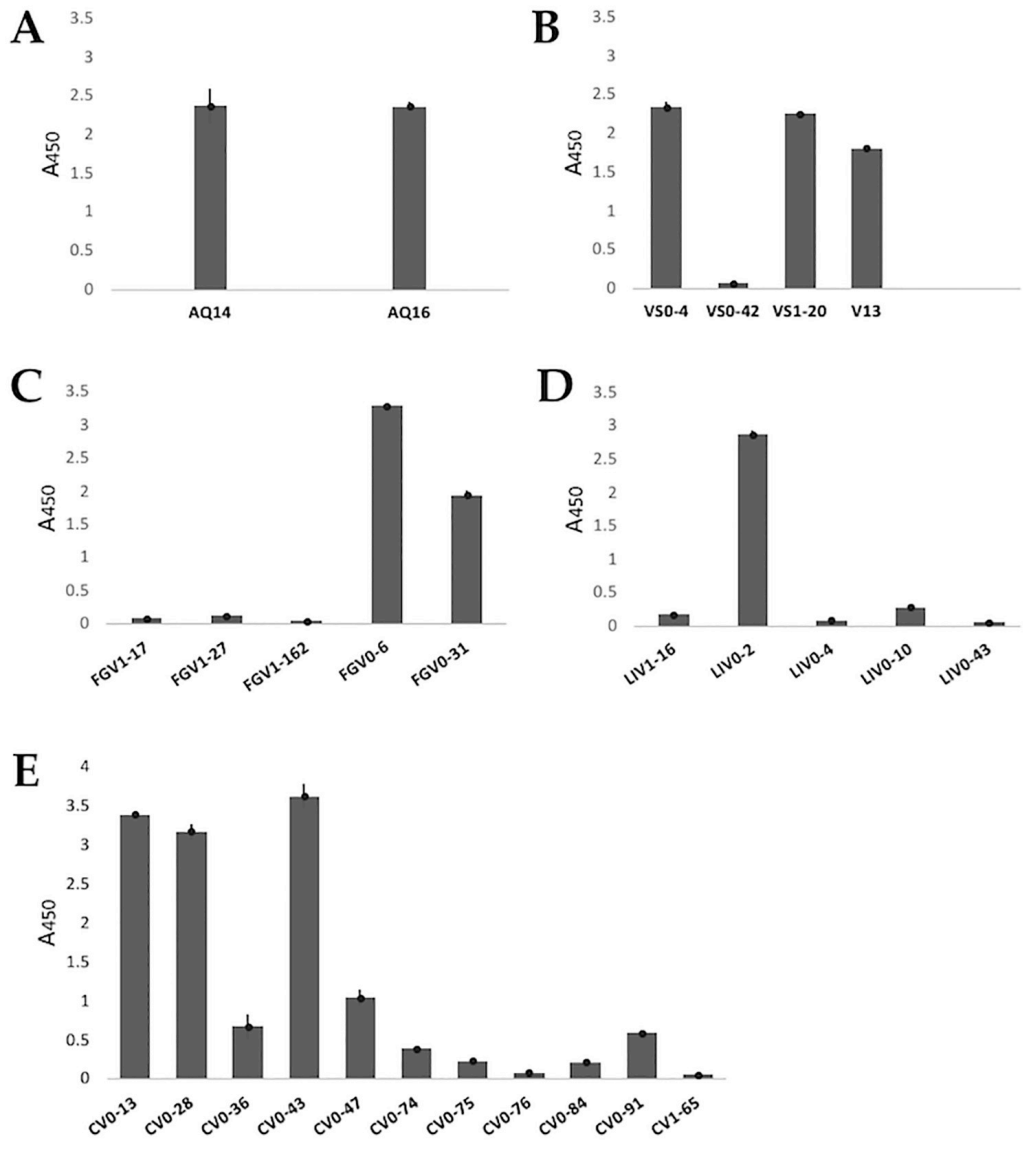
Recognition ability of the expressed antibodies by ELISA. The antibodies (A) anti-AQP1: AQ14 and AQ16; (B) anti-VEGF: VS0-4 and VS1-20, (V13 was used as positive control); (C) anti-FGF-2: FGV0-6 and FGV0-31; (D) anti-LIF: LIV0-2 and (E) anti-CEA: CV0-13, CV0-28 and CV0-43, showed positive recognition for their antigen. A450: Absorbance at 450 nm.

The antibodies that showed the highest levels of expression (with an absorbance at least twice that of the BSA which was used as negative control) were analyzed by ELISA, to evaluate and confirm their recognition ability to their antigen. As a negative recognition control, 1% BSA (protein used to block during panning) was used. Clones with an absorbance at least twice that of the negative control were considered positive. The antibodies with a positive recognition ability were: AQ-14 and AQ-16 anti-AQP1(Fig 5A); VS0-4 and VS1-20 anti-VEGF, V13 was utilized as positive control (Fig 5B); FGV0-6 and FGV0-31 anti-FGF-2 (Fig 5C); LIV0-2 anti-LIF (Fig 5D); CV0-13, CV0-28 and CV0-43 anti-CEA (Fig 5E); and GPV0-2 anti-GYPA.

**Fig 6:**
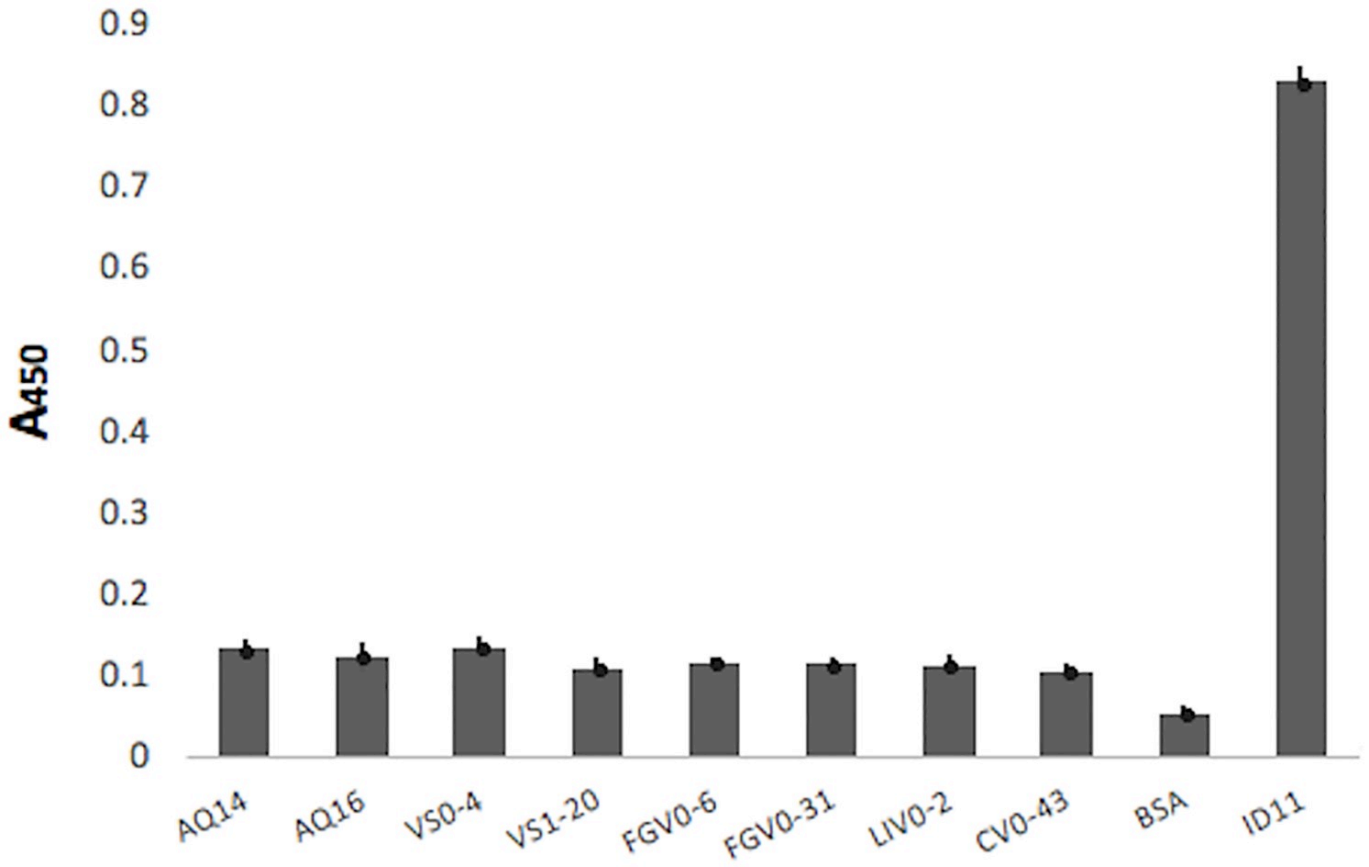
Cross-reactivity evaluation of vNAR antibodies with TGFβ. AQ14, AQ16, VS0-4, VS1-20, FGV0-6, FGV0-31, LIV0-2 and CV0-43 were used as capture antibodies in a sandwich ELISA to evaluate their binding to TGFβ, which is the target antigen of the frameworks used for the VS0 and VS1 libraries. The commercial anti-TGFβ ID11.16.8 mAb was used to detect the capture antigen. 1% BSA was used as a negative control. The anti-TGFβ ID11.16.8 mAb was used as a positive control, in an indirect ELISA format. None of the tested vNAR proteins gave a positive TGFβ binding response, the reactivity of the vNARs proteins were seven fold inferior to the reactivity of the positive control. A450: Absorbance at 450 nm.

To ensure that the clones were specific to their target antigen, their binding to TGFβ (target of clones T1 and T20) was evaluated by sandwich ELISA, since all the tested clones came from the VS0 or VS1 library. The efficacy of the binding was compared with the commercial anti-TGFβ ID11.16.8 mAb, carried out in an indirect ELISA format. None of the vNAR proteins tested gave positive TGFβ binding response, the reactivity of the vNARs proteins were seven fold inferior to the reactivity of the ID11.16.8 mAb used as positive control (Fig 6).

### Immunodetection of antibodies

**Fig 7:**
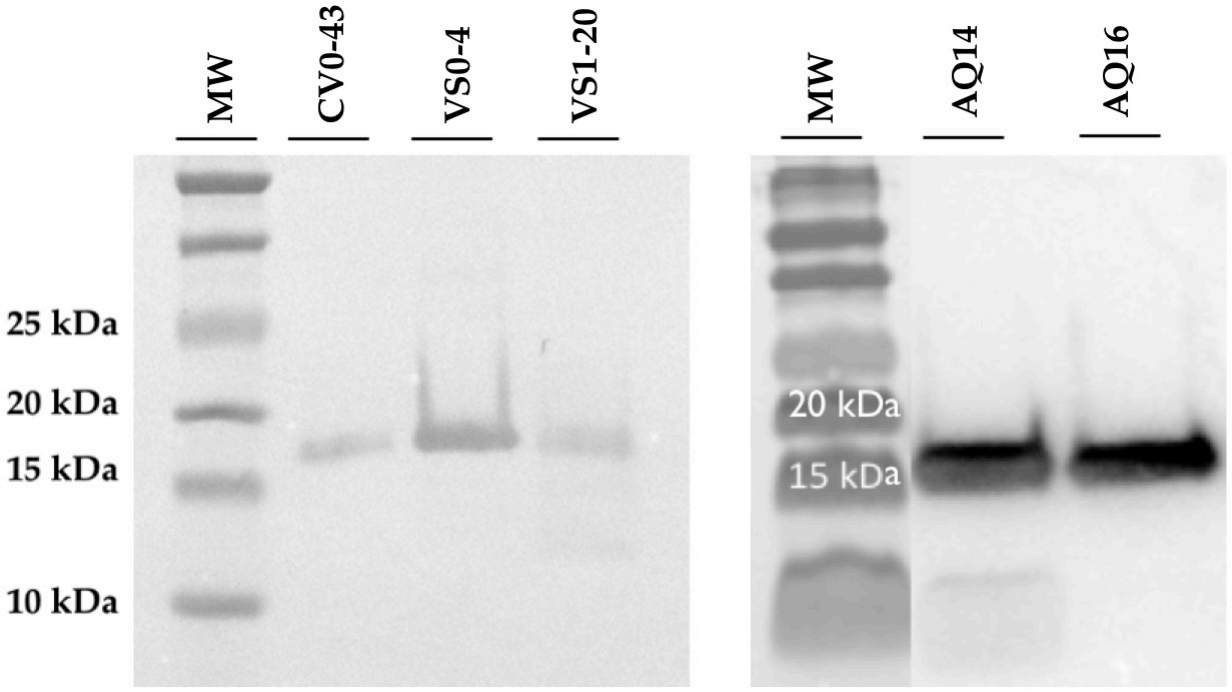
Western blot analysis of different antibodies. Western blot of the purified CV0-43, VS0-4, VS1-20, AQ14 and AQ16 vNAR. A commercial anti-HA-HRP antibody was used for the vNAR immunodetection. (MW) molecular weight marker.

Five vNAR antibodies, CV0-43, VS0-4, VS1-20, AQ14 and AQ16 that showed recognition by ELISA, were expressed in a larger volume and purified. Fig 7 shows the immunodetection results of the purified antibodies. In all the samples a band of the expected size was detected, corresponding to the molecular weight (theoretical) of the vNAR antibodies (~15 kDa including HA and His tails). The expression yields of the recombinant vNARs ranged from 0.1 to 0.25 mg/L.

### Three-dimensional *in vitro* angiogenesis assay based on endothelial cell spheroids

**Fig 8:**
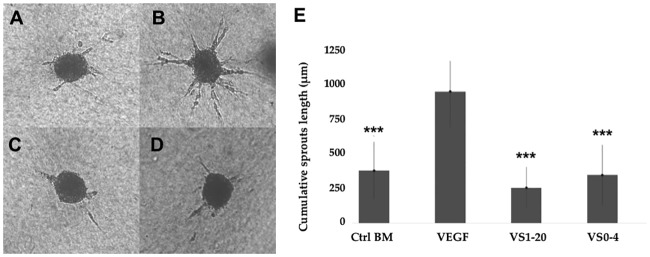
Three-dimensional *in vitro* angiogenesis assay based on collagen gel-embedded endothelial cell spheroids. Human umbilical vein endothelial cells (HUVEC) spheroids were treated with VEGF and anti-VEGF antibodies. The sprouts that originated from the spheroids were quantified digitally as described previously in the materials and methods. (A) Control BM; (B) EC + VEGF (C); EC+ VEGF + VS1-20; (D) EC + VEGF + VS0-4; (E) The cumulative sprouts length of capillary-like sprouts was measure after 24 h of incubation. The negative controls spheroids have a low basal sprout formation, that can be strongly stimulated by exogenous VEGF (50 ng/ml) and inhibited by treating the cells with anti-VEGF vNAR antibodies (10 μg at time 0 and 10 μg 4 hours later). Angiogenesis sprouts were significantly (P<0.001) inhibited when the spheroids were treated with VS1-20 and VS0-4 antibodies isolated from the synthetic libraries. BM (Basal medium).

The anti-angiogenic effect of the anti-VEGF antibodies from the synthetic libraries, was evaluated in an EC spheroid-based three-dimensional *in vitro* angiogenesis assay. The spheroids were stimulated with VEGF and treated with different anti-VEGF antibodies. The cumulative length of the sprouts was quantified after 24 h of treatment. The Fig 8 shows the results of the *in vitro* angiogenesis assay. VEGF (5 ng/mL) strongly stimulates the formation of angiogenesis sprouts originated from the EC spheroids (Fig 8B and 8E), up to three times the cumulative length of the sprouts from the control with no stimuli from VEGF (Fig 8A and 8E). When the spheroids stimulated with VEGF were treated with the anti-VEGF antibodies VS1-20 and VS0-4, a significant (P<0.001) inhibition of the sprouts was observed in comparison to the spheroids that were only treated with VEGF (Fig 8C, 8D and 8E). The treatment with VS1-20 presented the highest inhibition of the angiogenesis sprouts from the spheroids (74%), compared to treatment with VS0-4 (64%) and control treatment without stimulation with VEGF (60%).

### *In silico* analysis of anti-VEGF antibodies: Homology modeling, Docking, protein-protein affinity and interaction surface predictions

**Fig 9:**
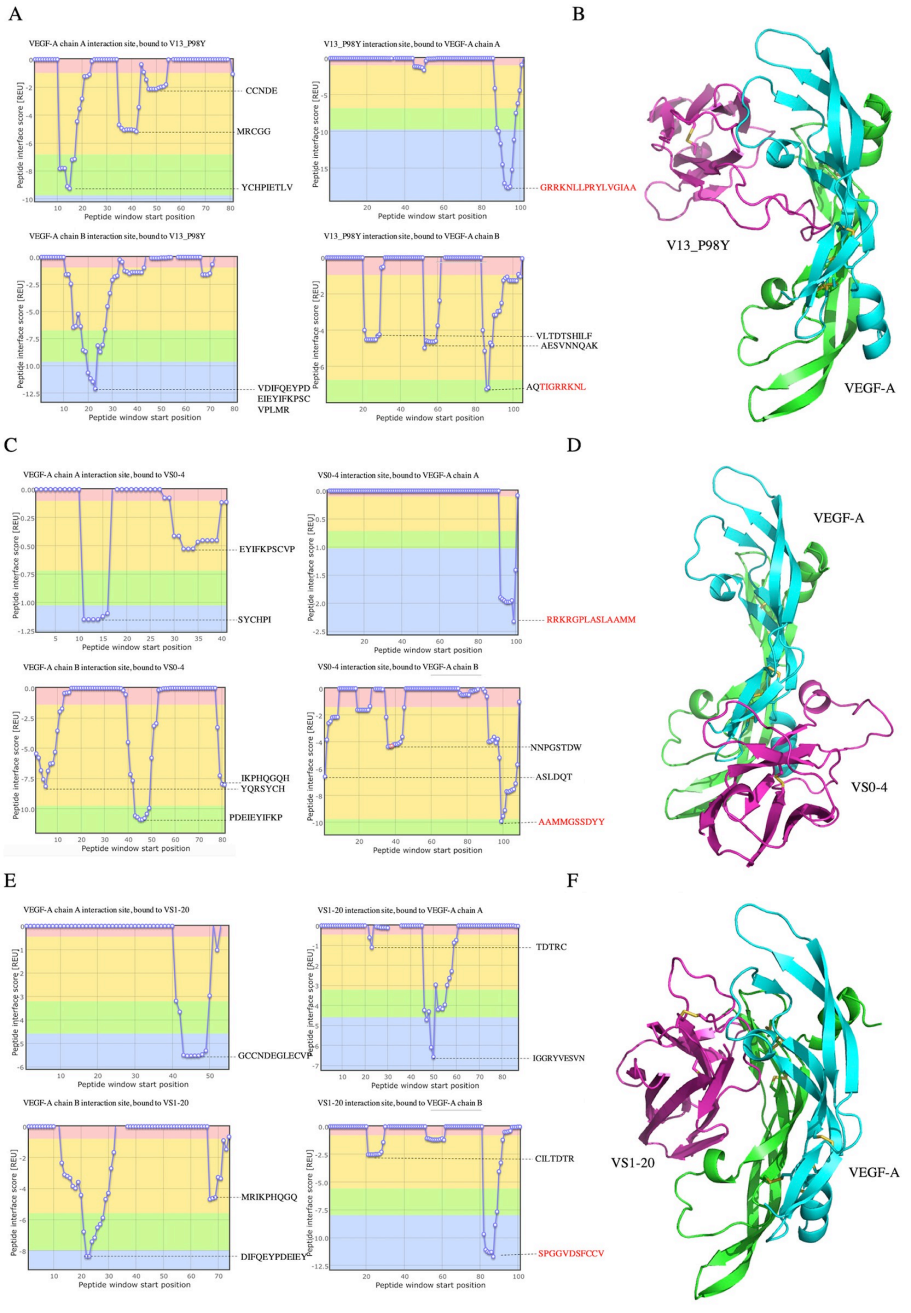
Protein-protein interaction analysis in ClusPro and Peptiderive web tool. (A) A plot of interaction regions of Positive control vNAR V13_P98Y with VEGF, in red the sequence of CDR3 of the vNAR. (B) V13_P98Y/VEGF complex, vNAR in magenta, and VEGF-A in green and cyan. (C) Interaction sites plot from Peptiderive of VS0-4/VEGF complex, in red the sequence of CDR3 of the vNAR. D) VS0-4/VEGF-A complex from ClusPro web tool. E) Peptiderive analysis of VS1-20/VEGF complex, indicate in red the interaction sequence of the vNAR CDR3 with VEGF. F) complex of VS1-20/VEGF.

The *in silico* models of VS0-4 and VS1-20 were obtained to predict the possible binding site of these antibodies to VEGF. We obtained five possible conformations along the dynamics (S1 and S2 Tables). The structures that had the longest existence time were selected. The refined three-dimensional models of vNAR showed a residue percentage of the most favored regions (A, B, L) for VS0-4 (89.3%), and VS1-20 (90.7%) (S3 Fig). Four possible interaction between the anti-VEGF vNARs (VS0-4, VS1-20 and V13_P98Y used as control) and VEGF were generated for protein-protein interaction analysis to identify the complex that best explains the binding between the vNARs and VEGF. A comparative analysis of the interaction energy of each model was performed (Table 3). In the control model V13_P98Y [24], an interaction score of -38.94 REU was identified. The CDR3 loop of V13_P98Y has the high score of interaction in the complex (Fig 9A and 9B). The complex VS0-4/VEGF-A obtained an interaction score of -29.97 REU. The interaction site of VS0-4 with VEGF was identified, the CDR3 loop is involved in union with VEGF chain A and B (Fig 9C and 9D). Meanwhile, the complex VS1-20/VEGF-A obtained an interaction score of -39.62 REU. The CDR3 loop of this vNAR interact with the chain B of VEGF-A (Fig 9E and 9F).

**Table 3:** vNAR/VEGF complexes interaction. The amino acids of the CDR3 that have the greatest contact with the VEGF molecule are shown in bold.

vNAR	Interaction site, bound to VEGF-A chain A	VEGF-A chain A interaction site	Interaction site, bound to VEGF-A chain B	VEGF-A chain B interaction site	Total Score (REU)
**V13_P98Y**	**GRRKNLLPRYLVGIAA**	YCHPIETLV	AQT**IGRRKNL**	VDIFQEYPDEIEYFKCVPLM	**-38.94**
**VS0-4**	**RRKRGPLASLAAMM**	SYCHPI	**AAMMGSSDYY**	PDEIEYIFKP	**-29.97**
**VS1-20**	IGGRYVESVN	GCCNDEGLECVP	**SPGGVDSFCCV**	DIFQEYPDEIEY	**-39.62**

### Blocking activity of VS0-4 and V13_P98Y on the VEGF/VS1-20 interaction

**Fig 10:**
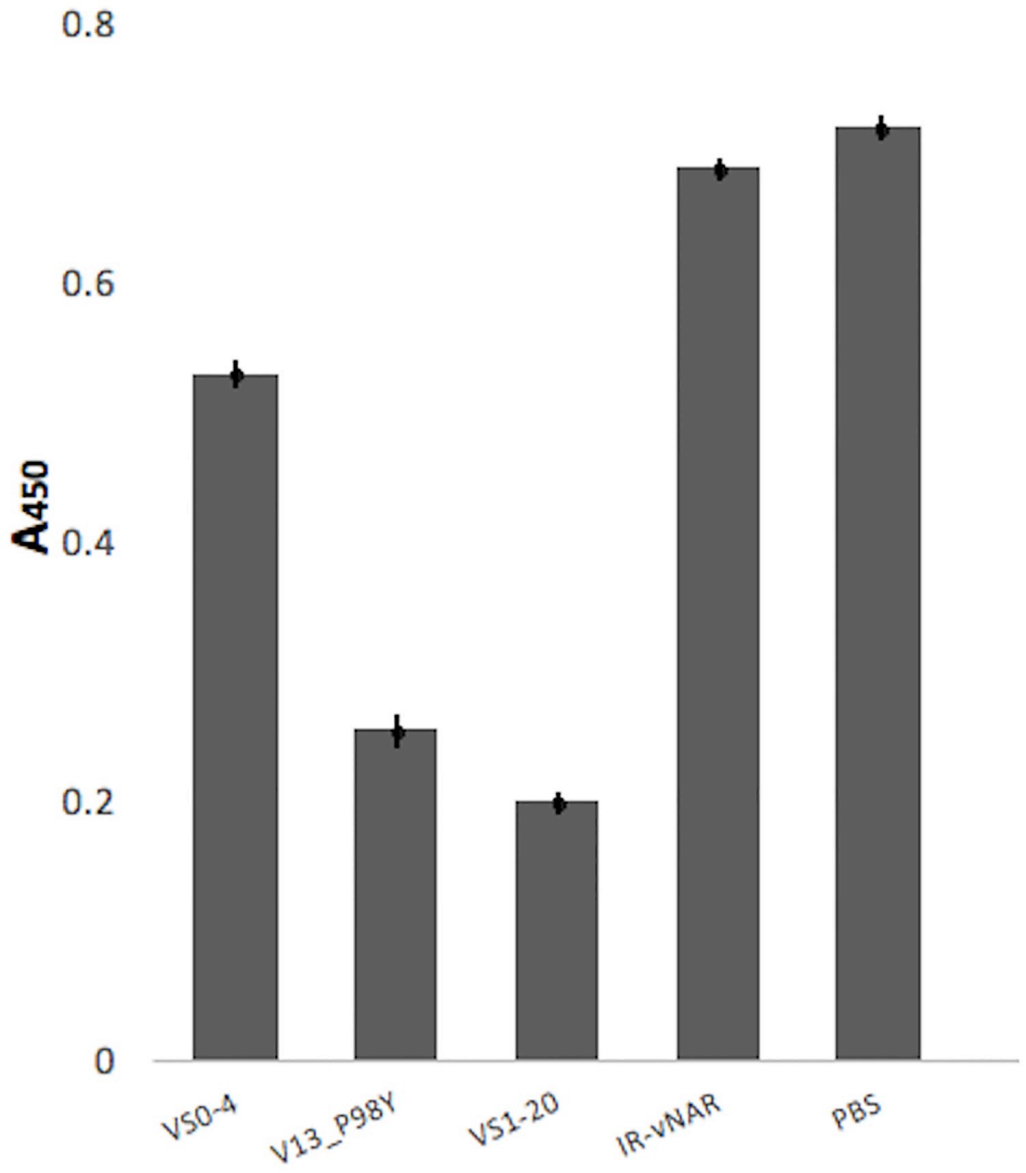
Binding ability of VS1-20-phage to VEGF by competitive ELISA. VS1-20-phage was tested to recognize VEGF in a competitive ELISA with soluble VS0-4 or V13_P98Y. Soluble VS1-20 was used as positive control. The irrelevant vNAR CV0-43 (IR-vNAR) was used as negative control. PBS control was used to represents 100% binding. The binding of VS1-20 was detected by an anti-M13-HRP antibody. When VS1-20-phage was tested to recognize VEGF in the competitive assay with soluble VS1-20, V13_P98Y, VS0-4 and IR-vNAR the signal reduction ranged between 75%, 65%, 25% and 5%, respectively, when compared with the PBS control. The greatest signal reduction was detected when soluble VS1-20 was evaluated. A450: Absorbance at 450 nm.

Since the *in silico* protein-protein interaction analysis predicted that VS1-20 recognizes an epitope very close to that of V13_P98Y, but distant to that of VS0-4 in the VEGF molecule (Fig 9), the results of the *in silico* analysis were tested by a competitive ELISA to evaluate whether VS1-20 and V13_P98Y have an overlapping epitope and could block one another’s binding to VEGF. Soluble VS0-4, V13_P98Y or VS1-20 were used to bind VEGF, and subsequently it was evaluated whether VS1-20 phage was capable of recognize an epitope available in VEGF or whether the soluble vNAR antibodies blocked the VEGF/VS1-20 phage interaction. Fig 10 shows the results of the signal reduction for each VNAR, as a negative control the vNAR CV0-43 (irrelevant vNAR) was used and a PBS control was also used which represents 100% binding.

When comparing the signal reduction of the soluble VS0-4, V13_P98Y, VS1-20 and the irrelevant vNAR with PBS control, VS1-20 shows the greatest difference in signal reduction (~ 75% of binding reduction) while V13_P98Y shows ~ 65% of binding reduction. Whereas testing VS0-4 showed ~ 25% signal reduction. Less than 5% signal reduction was observed when evaluating the irrelevant control vNAR.

## Discussion

Shark vNAR antibodies present special characteristics that made them attractive as therapeutic and diagnostic agents due to their small size, good tissue penetration, their long CDR3, as well as their thermic and chemical stability [9, 10, 12, 15]. It has been reported that the immune system of the shark offers a good antibody response against different proteins such as: HEL [14], Ebolavirus nucleoprotein [35], TNFα [36,37], ICOSL [38], VEGF [12], malaria PfHRP2 [39], amongst others. However, the IgNAR response of sharks is slower than the similar process observed in mammalian, and obtaining the desired antibodies could last up to 4–6 months [40, 41]. The generation and use of synthetic libraries has decreased the time at which an antibody can be isolated, since it avoids animal immunization [20, 21, 42].

In this work we present the construction of three synthetic vNAR antibody libraries, utilizing frameworks without Cys, with one and with two Cys within the CDR3 to reflect the number of Cys residues that occur naturally in the different types of vNAR antibodies [6, 8, 9, 16]. Only the CDR3 of the antibodies was diversified (conserving the number and position of Cys within this region). CDR3 has an especial interest because it is the CDR that forms the greater number of contacts with the antigen, it is localized in the center of the union site for the antigenic molecule and it is by far the most diverse loop in length and in sequence of the CDRs [43, 44]. Mutagenic forward oligonucleotides were employed due to the phagemid vector pComb3X utilized, that has a negative replication origin.

The sizes of the obtained libraries were 1.8x10^9^, 6.7x10^9^ and 5.7x10^11^, for VS0, VS1 and VS2 respectively; the variability percentage was up to 70%. The efficiency of the mutagenesis was according to the expecting, considering that the technology of Kunkel mutagenesis offers efficiencies that range from 50–90% [45]. Other works report synthetic library sizes of single domains that range from 1x10^8^-3x10^9^ [20, 22, 46–48]. Our libraries presented good diversity with a moderate recombinant repertoire. To improve the efficiency of selection of the clones obtained by mutagenesis, the sequences of the three frameworks were synthesized including three stop codons (no amber) after the canonical Cys of framework 3. The mutagenic oligonucleotides were designed to eliminate these three stop codons. A successful alignment and extension resulted in the proper display of the protein on the surface of the phage. In this way, when covering an ELISA plate with an antibody against the HA tag, that is in the forward direction to the vNAR domain sequence, only the clones that display a complete vNAR on the surface of the phage, in which the mutagenesis occurred correctly, could be identified using an anti-M13-HRP antibody. Under this principle, 92 clones randomly selected were evaluated. The percentage of phages that displayed a complete vNAR was approximately 86%. In conjunction, these results confirm that within the libraries there is sequence diversity, with a high frequency of complete displayed vNARs.

The validation of the libraries was conducted by selection against six different mammalian proteins: AQP1, VEGF, FGF-2, LIF, CEA and GYPA. The selection in all cases was conducted in four rounds, incrementing the selection rigor during each round. At least one functional antibody was found against each tested antigen utilizing the library VS0. For the VS1 library functional antibodies were detected against VEGF, additionally other antibodies of this library were found against the rest of the antigens, however they did not present detectable expression. The employment of the VS2 library during selection did not yield any functional antibody. With the present format, the used *E*. *coli* strain, insert a Gln instead of the amber codon, further work needs to be conducted with all the isolated clones from this library to determinate if these clones are functional. Clones with positive recognition to their target antigen were evaluated for binding to TGFβ, the target molecule of T1 and T20 clones, used as the frameworks for the VS0 and VS1 libraries, respectively. None of the tested vNARs gave a positive TGFβ binding response, compared to the reactivity of the commercial ID11.16.8 mAb used as a positive control. This suggests that there is no cross-reactivity of the evaluated vNARs, with the original binding antigen of the clones used as frameworks for the libraries.

During selection of the three libraries, some antibody sequences were obtained that included an amber stop codon. As mentioned, this codon is partially suppressed in some strains of *E*. *coli*, such as TG1 and ER2738 [49]. For the validation of the libraries, we worked with clones that did not present an amber stop codon. The utilization of posterior codon substitution steps for a Gln, such a PCR with primers that modify this codon or chemical synthesis of antibodies, could be used to evaluate more antibody sequences.

Up to this point, the VS0 library from the framework without Cys at CDR3 suggested the best selection results. The T1 framework used for the construction of this library, belonged to the type IV vNAR, without Cys in the CDR3 and two canonical Cys in positions 22 and 83 (FR 1 and FR3, respectively) [16]. For the construction of VS1 library, a type II vNAR was used (T20 framework). Selection with VS1 also resulted in antibodies against all antigens, but less clones than VS0 were selected, and not all the antibodies were evaluated due to expression problems or the insertion of an amber codon.

Interesting results were obtained with VS2 library. This was the largest library although it possessed less diversity (60%). For this library, a framework with two Cys within the CDR3 was used, this however, is not in accordance with the type I vNAR antibodies (which has only been reported in the nurse shark (*G*. *cirratum)* [9]. We describe it for the first time in this work and we propose it as type V vNAR. It is known that vNAR antibodies, have a higher frequency of polar and charged amino acids in the regions exposed to the solvent, which correspond to the VH/VL interface of the conventional antibodies, this makes them highly soluble in water [7, 50]. However, mutagenesis does not prevent the appearance of hydrophobic amino acid residues as well as additional Cys [47]. We speculate, that having the longest CDR3 of the three libraries, with two invariable Cys, plus the possibility of the appearance of hydrophobic amino acids (and new Cys residues) that are not natural occurring in this region, may have resulted in incorrect folding of the domains. This could explain the lack of functional antibodies in this library. Al Qaraghuli and Ferro [51] evaluated different crystalized single domain antibodies in response to various antigens that included type I and type II vNAR antibodies and report that each type of vNAR contains a unique conformation of the CDR3 (i.e. pleated type I vNAR or extended type II vNAR). This tightly packed type I vNAR allows penetration into the active sites of different targets, as has been shown for enzymes, for which active site binding and functional inhibition by vNARs have been reported [14]. Based on this, we could hypothesize that the library VS2 which includes two Cys in the CDR3 as the type I vNAR, could face antigens with other structural characteristics to those evaluated in this work.

The amount of Cys residues within the CDR3 of the antibodies often generates concern and the studies of this amino acid in the CDR3 of the vNAR antibodies are yet rare. Furthermore, some synthetic libraries have been developed intentionally avoiding the appearance of Cys within the CDR3 [22, 47, 48]. Our results suggest that vNAR isolated from synthetic libraries lacking Cys in the CDR3, are more easily selected than those vNAR with one or two Cys in the CDR3, this might be due to a more flexible topology and less restrains for the paratope [52]. It was observed that having a library size greater than 1x10^11^ does not ensure the success of the library as our results show with VS2 library.

Some selected antibodies against VEGF, CEA and AQP1 were used for immunodetection and expression in larger volumes. The evaluated protein presented an expected size of approximately 15 kDa. The expression levels were low with yield around 0.25 mg/L. Other studies report expression levels of recombinant vNAR antibodies ranging from 0.07 mg/L to 4 mg/L [15, 20, 36]. The pComb3X vector utilized is a phagemid and not an expression vector, therefore a change in this system could result in higher yields. It is also known, that the secretory efficiency of recombinant proteins is usually low when secretion signals are used [53], as we observed in our study. The expression of recombinant proteins is always a challenge and different conditions should be evaluated with the object of increasing the expression levels.

To evaluate whether the antibodies isolated from the synthetic libraries were functional, the antibodies against VEGF were selected. VEGF is a model antigen in our research group and to date anti-VEGF antibodies have been isolated from immune libraries [12] and antibodies have been developed whose VEGF neutralization has been improved through *in silico* modeling [24].

The *in vitro* anti-angiogenic effect of VS1-20 and VS0-4 anti-VEGF antibodies was measured in an EC spheroid-based three-dimensional *in vitro* angiogenesis assay, which mimics the formation of angiogenesis *in vivo*, through the stimulation of the EC with VEGF [54, 55]. In these assays, the EC are cultivated in a collagen and metocel matrix that stimulate the union, the migration and the rapid formation of the EC tubes, reflecting this event *in vivo* [56]. The results of the angiogenesis assay, showed that the stimulation of EC with VEGF strongly induced the formation of sprouts derived from the spheroids. These angiogenesis sprouts were significantly inhibited when the spheroids were treated with antibodies isolated from the synthetic libraries (P<0.001). Of the clones tested VS1-20 showed the greatest inhibition of VEGF-induced angiogenic sprouting, with a 74% less cumulative length of the sprouts in comparison to the control without anti-VEGF treatment.

The three-dimensional structures of anti-VEGF antibodies (VS0-4 and VS1-20) were generated by *in silico* modeling. The refined three-dimensional models of vNARs showed a residue percentage of the most favored regions (A, B, L) for VS0-4 (89.3%), and VS1-20 (90.7%). A good quality model would be expected to have over 90% in the most favored regions [31]. Docking, protein-protein affinity and interaction surface predictions of anti-VEGF antibodies, suggested that VS1-20 and VS0-4 recognize different epitopes, whereas VS1-20 and V13_P98Y recognize very close epitopes in the target molecule. The *In silico* analysis suggested that VS1-20 has a greater interaction with VEGF than VS0-4 and even than the control antibody V13_P98Y, an *in silico* maturated antibody with an *in vitro* and *in vivo* anti-angiogenic effect previously reported [24]. The results of the *in silico* analysis were supported by a competitive ELISA, it was evaluated if VS1-20 and V13_P98Y have an overlapping epitope and could block the union each other to VEGF. When VS1-20-phage was tested to recognize VEGF in a competitive ELISA with soluble VS0-4 or V13_P98Y a 25% and 65% signal reduction, respectively, was observed. As expected, when evaluating soluble VS1-20, the greatest reduction in the signal was shown, since it binds to the same site as VS1-20 phage. A signal reduction of less than 5% was observed when evaluating the negative control vNAR. Similar results of the blocking activity of V13_P98Y and VS1-20 were observed (65% and 75%, respectively). These results suggest that VS1-20 and V13_P98Y have very close or overlapping epitopes, which could block each other’s binding to the VEGF molecule, in accordance with our *in silico* observation.

Additional to the synthetic libraries that were presented, this work also reports the existence of a vNAR type with different Cys pattern to all previously reported types. Different clones of this type (S2 Fig) have been isolated at least 10 times in our laboratory (e.g. TN16 and V32R with patent number: PCT/ES2014/070332) [57], from the natural immune repertoire of our shark model *H*. *francisci*. This vNAR, that we propose as type V, is similar to type I, with a pair of Cys in the CDR3. However, it does not present non-canonical Cys in the FR2 and FR4 that are characteristic of the type I [6, 9], instead it possesses a pair of non-canonical Cys in the CDR1 (positions 29 and 32). The CDR3 loop of this type presents lengths up to 24 residues (with an average length of 16 aa). More studies are needed to elucidate the arrangement of Cys bond pattern of the proposed vNAR type V.

In conclusion, in this work, the selection against the different antigens, resulted in a greater number of clones when using the library constructed from a framework without Cys. This could suggest that the use of vNAR frameworks without Cys residues could increase the isolation of functional antibodies within the synthetic library. This is the first report where frameworks with different Cys numbers are evaluated to determine how this can alter the isolation of preferred clones from the synthetic libraries in order to get new drugs. In addition, the results of the *in silico* analysis of anti-VEGF antibodies suggest that antibodies isolated from synthetic libraries can rival antibodies whose affinity has been improved through *in silico* modeling.

## Supporting information

S1 Fig Mutagenic double-stranded DNA. T20 Uracilated single-stranded DNA (ssDNA) noted with a black arrow, was converted into larger mutagenic double-stranded DNA (dsDNA). The desired product is marked with a red arrow, which represents the correctly extended and ligated dsDNA. dsDNA has a lower electrophoretic mobility than ssDNA. 1% agarose gel stained with ethidium bromide.
(TIF)

S2 Fig Sequences of vNAR domains with Cys pattern corresponding to the proposed type 5. Sequences possesses a pair of non-canonical Cys in the CDR1 and an additional pair of Cys in the CDR3. The Cys in the CDR1 remain in the same positions (29 and 32). The different regions of the vNAR are labeled. FR: Framework, CDR: complementarity-determining region, HVR: Hypervariable region. Canonical Cys are enclosed in red. Non canonical Cys are enclosed in blue.
(TIF)

S3 Fig Refined three-dimensional models of VNAR, and Ramachandran plot. (A) VS0-4. (B) VS1-20.
(TIF)

S1 Table Three-dimensional structures of VS0-4. Five possible conformations were predicted by homology-based modeling and refined by molecular dynamics.
(DOCX)

S2 Table Three-dimensional structures of VS1-20. Five possible conformations were predicted by homology-based modeling and refined by molecular dynamics.
(DOCX)
