# Supplementary figures and images for: Synthetic libraries of shark vNAR domains with different cysteine numbers within the CDR3
Source: PLoS One. 2019 Jun 17;14(6):e0213394. doi: 10.1371/journal.pone.0213394 (PMC6576789; doi:10.1371/journal.pone.0213394)

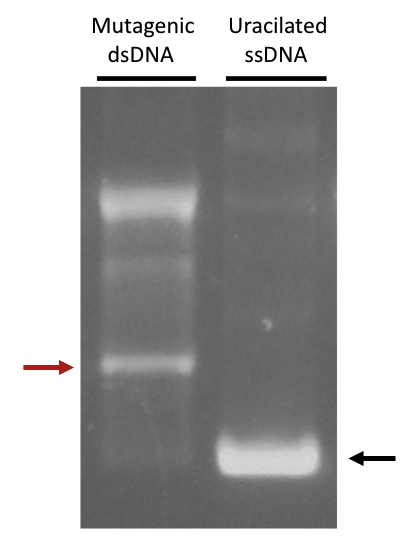

Supplement: S1 Fig — T20 Uracilated single-stranded DNA (ssDNA) noted with a black arrow, was converted into larger mutagenic double-stranded DNA (dsDNA). The desired product is marked with a red arrow, which represents the correctly extended and ligated dsDNA. dsDNA has a lower electrophoretic mobility than ssDNA. 1% agarose gel stained with ethidium bromide. (TIF) [file pone.0213394.s001.tif]

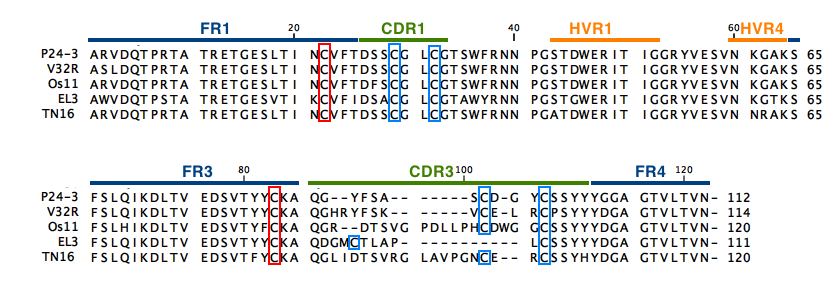

Supplement: S2 Fig — Sequences possesses a pair of non-canonical Cys in the CDR1 and an additional pair of Cys in the CDR3. The Cys in the CDR1 remain in the same positions (29 and 32). The different regions of the vNAR are labeled. FR: Framework, CDR: complementarity-determining region, HVR: Hypervariable region. Canonical Cys are enclosed in red. Non canonical Cys are enclosed in blue. (TIF) [file pone.0213394.s002.tif]

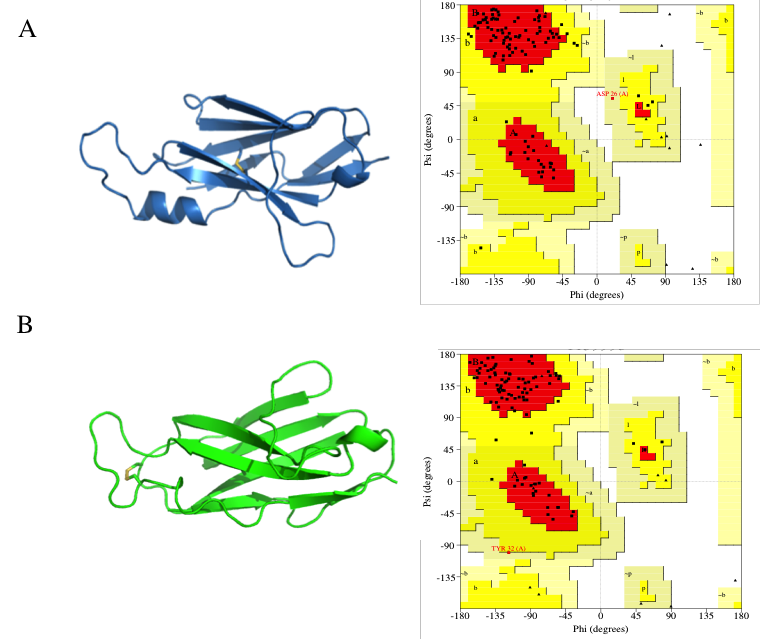

Supplement: S3 Fig — (A) VS0-4. (B) VS1-20. (TIF) [file pone.0213394.s003.tif]
