# Supplementary material for: Synthetic libraries of shark vNAR domains with different cysteine numbers within the CDR3
Source: PLoS One. 2019 Jun 17;14(6):e0213394. doi: 10.1371/journal.pone.0213394 (PMC6576789; doi:10.1371/journal.pone.0213394)
Supplement: S1 Table — Five possible conformations were predicted by homology-based modeling and refined by molecular dynamics. (DOCX) [file pone.0213394.s004.docx]

**S1 Table. Three-dimensional structures of VS0-4.** Five possible conformations were predicted by homology-based modeling and refined by molecular dynamics.

| Conformation | | Number of structures | Existence time (ns) |
| --- | --- | --- | --- |
| 1 | 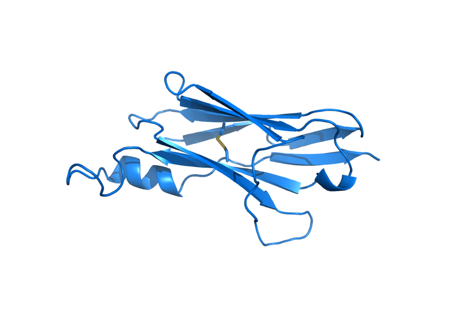 | 52 | 1.04 |
| 2 | 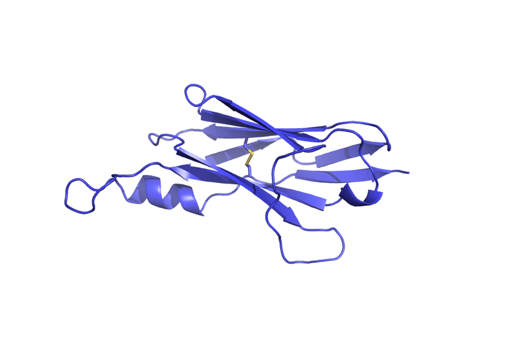 | 759 | 15.18 |
| 3 | 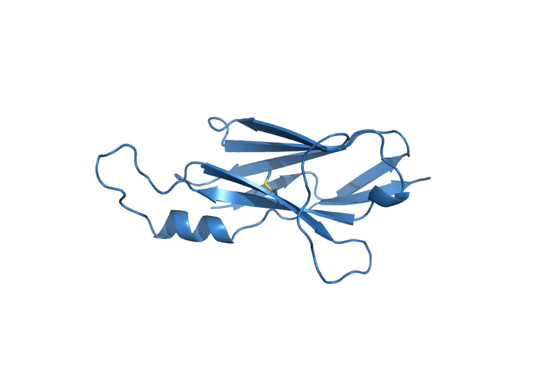 | 1348 | 26.96 |
| 4 | 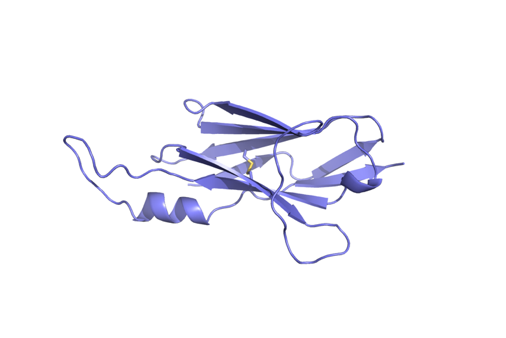 | 290 | 5.8 |
| 5 | 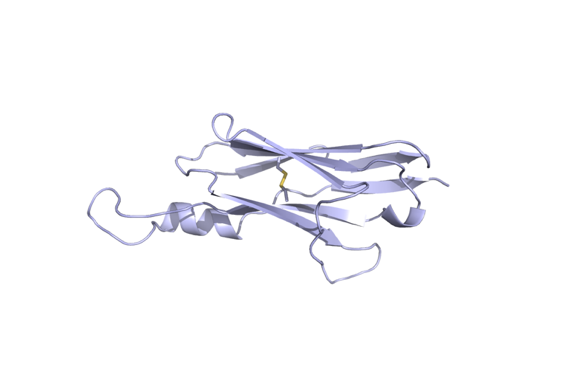 | 51 | 1.02 |
